# Neither entrepreneurship nor intrapreneurship: a review of how to become an innovative split-off start-up

**DOI:** 10.3389/fsoc.2023.1267706

**Published:** 2023-09-25

**Authors:** Christina Elisabeth Knossalla, Claus-Christian Carbon

**Affiliations:** ^1^Department of General Psychology and Methodology, University of Bamberg, Bamberg, Bavaria, Germany; ^2^Research Group EPÆG (Ergonomics, Psychological Æsthetics, Gestalt), Bamberg, Bavaria, Germany

**Keywords:** entrepreneurship, corporate start-up, intrapreneurship, split-offs, spin-offs, innovation, leadership

## Abstract

Splitting off departments from corporations in order to establish corporate start-ups has become of strategic importance for the performance and innovation of corporations. While the settlement process is widely practiced, there is a lack of knowledge of how entrepreneurship may exist in such split-offs. The main aim of this study was to explore how entrepreneurship in corporate start-ups can exist in order to contribute to corporate performance. Based on a systematic literature review from 2021 to 2023, which resulted in a total of 1,516 scientific, English-language articles in economic journals, a total of 150 articles were analyzed in-depth. Our research shows that it is of crucial importance that corporations position leaders with an appropriate mindset and behavior at all levels as early as starting the split-off process, which is, however, neither entrepreneurship nor intrapreneurship. The niche corporative start-up area shows that entrepreneurship is a continuum and requires a new definition of corporate start-up entrepreneurship (CSE). For corporate start-ups to be successful, we revealed that there needs to be (1) the appropriate legal form, which ensures ownership but also the risk of the leaders, (2) an explorative business rather than exploitation, (3) variable compensation rather than fixed and (4) corporate entrepreneurs rather than employees and managers. Implications of the findings for entrepreneurial leadership theory development and future research are discussed.

## Introduction

Fastly growing start-ups that get to the heart of customer requirements pose a formidable challenge for existing corporations. On the one hand, there are start-ups, which, due to their small independent organizational form, can quickly adapt to the ever-changing market and customer requirements, bring innovations to the market quickly, thus being fast in execution and using new technologies, and ultimately take a large market share from established corporations (Mishra, 2020). On the other hand, large corporations struggle due to their entrenched structures, often struggling with change, rigid organizational structures with numerous, settled employees, being slower in execution than their start-up competitors, proceeding business models that are largely related to exploiting working business models and products rather than exploring new, innovative business areas. Start-ups are the biggest competitors for corporations that try to be innovative (Pfennig, 2021). As a result, organizations split up individual divisions of the corporation to create the fictitious space of a start-up under the umbrella of the parent corporation (= corporate start-up; Dalton and Dalton, 2006). What they neglect through pure organizational reorganization of split-offs is Schumpeter’s findings from 1912: Entrepreneurial innovation is central to economic development and the driving force for competitiveness. However, innovation does not come from growth in the context of core business. This is because concentrating on the core business brings optimization up to a certain point but excludes the possibility of releasing resources to devote to research and development and, thus, to innovation. Furthermore, in the innovation process, the entrepreneur is the central innovator because they are the only ones carrying out innovation intelligently, risk-taking, breaking up old and creating new traditions by shareholding their ideas (Schumpeter, 1912). In the split-off process, corporations fill their leadership positions with managers already hired in the corporation rather than with hiring entrepreneurs. These ambitions to introduce entrepreneurship into corporations lead, at most, to intrapreneurship but not entrepreneurship. This leads to the fact that introducing innovation fails, and all efforts are short-lived with relatively little seriousness (Mishra, 2020). The research for reliable figures regarding the success of corporate start-ups shows that articles provide widely diverging success values or that no figures are provided at all. What is uniform, however, is the statement that business start-ups must be carried out with better performance (Jung et al., 2015; Hundt, 2018). Concerning start-up success in general, the 3–30 rule is often applied: within 3 years, 30% of all newly founded businesses terminate their business activities (Held, 2019).

The importance of entrepreneurship in new venture creation (e.g., start-ups) and the effect on innovation and economic success is confirmed widely (Baron, 2007; Sambasivan et al., 2009). However, many scientists focus on *intra*preneurship, which cannot lead to innovation (see Schumpeter). What is missing scientifically and in practice is which antecedents for corporate start-up entrepreneurship (CSE) must be given so that it can prevail in this niche organizational framework and then, in turn, pay off in terms of innovation. Accordingly, the main objective of this article is to enhance the understanding of how entrepreneurship may exist in a corporate start-up context. Therefore, our first requirement is to define a uniform framework for what can be understood by the term corporate start-up. To date, there has been no uniform definition of this. The second requirement is to derive corporate start-up entrepreneurship (CSE), which is needed for innovation in split-offs. We did so through a comparison between entrepreneurship and intrapreneurship. A descriptive research approach was chosen for this purpose. Our third requirement is to assess the antecedents for CSE based on the conceptual model for corporate entrepreneurship. We developed our line of argument on a model that has been derived by Urbano et al. (2022), giving an overview of individual, organizational and environmental antecedents for CE but with a broader perspective of new ventures as well as no new business additions (Urbano et al., 2022). All three requirements contribute to a more transparent view of how corporations can achieve innovation with corporate start-ups as new ventures. We elaborate on the current research status of the fields: start-ups, corporate start-ups, *entre*preneurship, and *intra*preneurship. With this, we contribute to enhancing the understanding of how entrepreneurship may exist in a corporate start-up context and which antecedents have to be established when splitting off.

To answer the research question of which organizational antecedents have to be established to create CSE in corporate start-ups, this study is organized in five main sections. The first section focuses on the conceptual model (1) which was used to structure the systematic literature review (SLR). We then described the method (2) and steps used in our SLR. In our results (3) section we delineate the framework and the specifics of split-offs from corporate groups, which shall be referred to as corporate start-ups and external start-ups, to derive the particular context that plays an essential role concerning corporate entrepreneurship. Similarities and differences between corporate and external start-ups are highlighted in the result section to clarify what to look out for when entrepreneurship is to be achieved in corporate start-ups. Moreover, the results of the SLR show a comparison of entrepreneurship and intrapreneurship in order to derive corporate start-up entrepreneurship thereupon. Finally, the limitations and implications of the conclusive results are discussed (4) and concluded (5).

### Conceptual model

**Figure 1:**
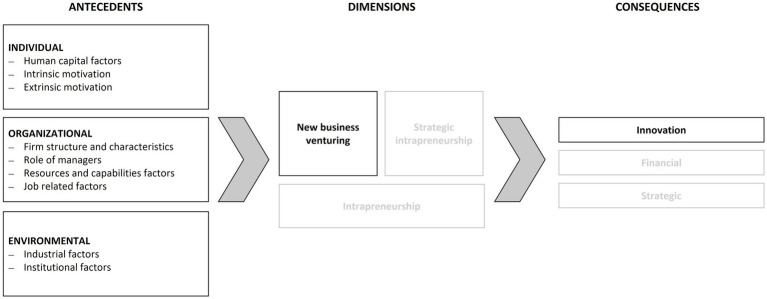
Conceptual model for corporate entrepreneurship in new business venturing (own representation based on Urbano et al., 2022).

To close the research gap regarding what is CSE and which antecedents must be given in advance we adapted the conceptual model for corporate entrepreneurship from Urbano et al. (2022) (see Figure 1). The rationale behind this is, as far as we know, it is one of the latest SLR results focusing on all three levels of antecedents, individual, organizational and environmental, with a ground basis of literature. With our understanding of entrepreneurship aligning with the findings from Schumpeter, the adaption of the model for our investigation is necessary, as we understand entrepreneurship is solely possible with new business venturing plus, in this research, we focus on the niche of corporate start-ups. For the sake of completeness, Urbano et al. also mention the dimensions of strategic intrapreneurship (no new business addition but rather restructuring and organizational rejuvenation) and intrapreneurship, in addition to the dimension of new business venturing. Both dimensions are out of focus for our work and, therefore, removed from the original model, as shown below. Lastly, the consequences of new business venturing shall be innovation and not, as Urbano et al. put it, strategic and financial. Nevertheless, the adapted model allows to provide a systematic content analysis and organizing framework for the SLR we undertook.

### Definition of new business venturing

In the present paper, we understand new business venturing as adding new (innovative) business to the firm by growing organically (Harzing, 2002). Organic growth is defined as prosperity resulting from within and with internal resources (e.g., increasing sales or service volume or broadening the customer range; Dalton and Dalton, 2006). Organic growth can be divided into three areas: invest, meaning to reallocate resources to a focus area; perform, which means to excel at commercial functions; and create, which is understood as creating new products or services. As we focus on split-offs, we address the lever of create. This lever is understood as achieving innovation when a certain level of growth and stability prevails, making the most significant difference in outperforming the competition (Ahuja et al., 2019). In this paper, focusing on split-offs, we do not consider inorganic growth, which is defined as external business growth (e.g., acquisitions, takeovers, or mergers; Dalton and Dalton, 2006) or any hybrid growth strategies blending the previously mentioned two and meaning that in parts resources are borrowed or purchased from the market and in portions growth is achieved from the corporation’s own resources (Agnihotri, 2014).

### Definition of innovation

We understand innovation along the classic definition of Schumpeter as “doing of new things or the doing of things that are already done in a new way” (Schumpeter, 1947, p. 14), which includes the creation of a new product, a new production method, the development of a new market, a new source of raw materials or a new organization. As a prerequisite, he states that the innovators are entrepreneurs who must design and implement the new thing themselves. Thus, they are a shareholder of the innovation and willing to take risks since they are entering the new and uncertain (Schumpeter, 1912).

## Methods

### Data collection

**Figure 2:**
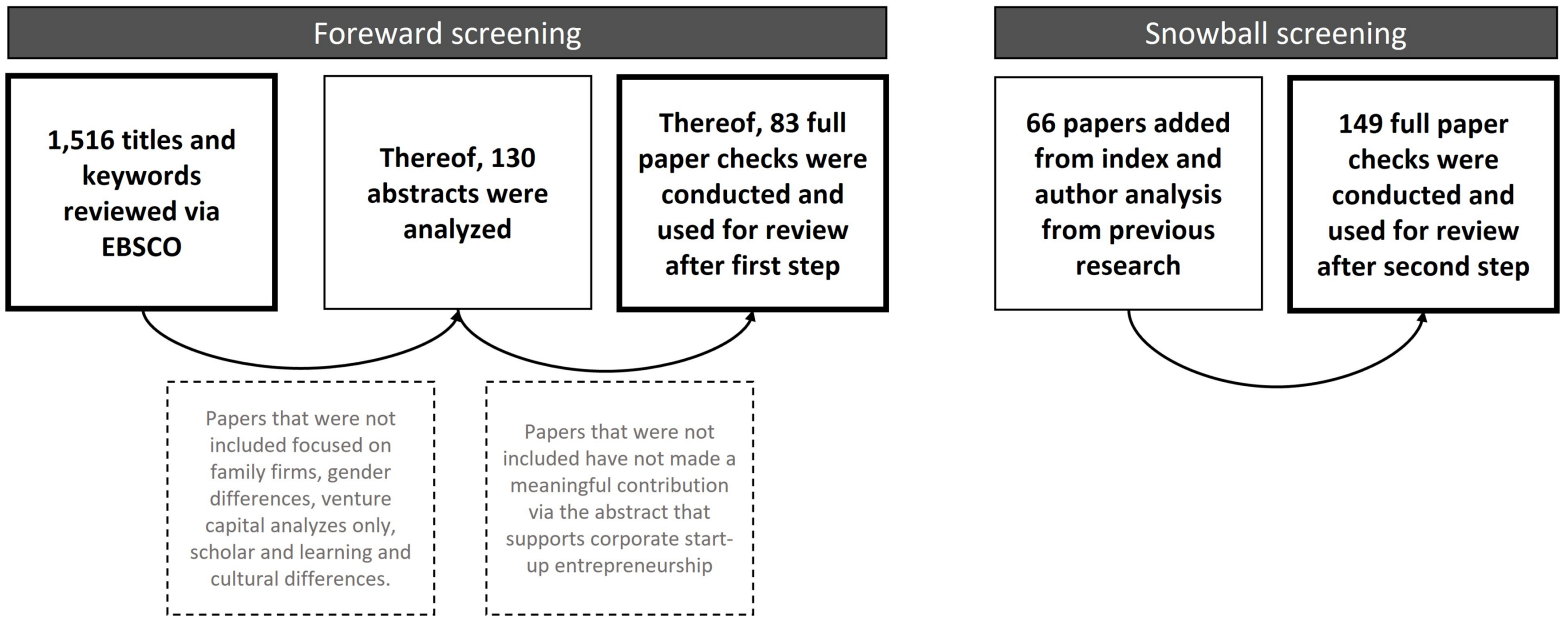
Data collection and article selection process (own representation).

Through a systematic literature review (SLR), including the steps of Tranfield et al. (2003), this study aimed to gain deeper insights into the impact of original entrepreneurship as a leadership style in corporate start-ups. The literature search was performed through two main procedures displayed in Figure 2: preliminary data analysis through forward database screening and secondary screening through a snowball systematic. We started with identifying the most relevant keywords of the research area, proceeded with the selection of studies, assessment of the quality of the papers, data extraction and concluded with the data synthesis as a result.

The first analysis (forward screening) is based on EBSCO database and took place between 2021 and 2023. EBSCO was chosen due to its coverage of articles that focus on management and new venture topics. Supplementary Tables S1a, S1b, show which keywords were used to start the elaboration. We decided on the following keyword combinations as there are the most commonly used terms in the literature to describe entrepreneurship in corporate start-ups: “corporate start-up,” “corporate start-ups and entrepreneurial leadership,” “corporate start-ups and intrapreneurship,” “corporate split-off” and “corporate split-off leadership.” For each keyword combination, it can be deduced how many articles were searched. A combination of keywords had to be chosen, as the individual terms would have yielded too many results in terms of quantity and too many deviating results in terms of content. We used Boolean logic (using “AND” or “OR”) to connect the keywords. The combinations consisted of one term from the organizational setting and one term from the area of entrepreneurship in order to link the two research areas. We searched for these words in the title, abstract, keywords, and in the body text and did not limit our search to any specific period of time.

*Leadership: initially, entrepreneurial leadership was included in the search. However, in the course of the research, it became apparent that the term leadership was too generalist, and too many results were obtained. Accordingly, the focus was subsequently placed only on entrepreneurship.

The trustworthiness of the research findings was ensured by focusing on scientific, peer-reviewed work only. Therefore, only a filter was applied to all search engines to display peer-reviewed publications. A further filter was set on the language setting, English and German only, due to the limited authors’ linguistic expertise.

The combinations yielded 1,516 articles via the EBSCO platform. All of these articles were further analyzed for titles and their corresponding keywords. If the title and keywords matched the topic of the study, the abstract was analyzed in a further step. Articles that did not focus on entrepreneurship/intrapreneurship in corporations were excluded. These were articles focusing on family firms, gender differences, venture capital analyses only, scholar and learning and cultural differences. One hundred thirty articles were further analyzed concerning the relevance of their abstract. Relevant, matching research papers were then stored in our reference managing software for further and detailed review. The sum of the saved and pre-screened papers corresponds to a total amount of 83 papers.

The second analysis, a snowball search systematic, was applied to identify the most recent and most cited literature based on the previously systematically reviewed literature. The most recent and directly linked literature was identified through a related search for relevant most cited authors. For this purpose, the literature indexes from the 83 articles of our first step of SLR were screened, and the frequency of author mentions was analyzed. Thus, another 87 articles were added for a full paper check analysis. Supplementary Table S2 identifies the authors who were previously cited more than once among the 83 articles. The second part shows which authors and papers have emerged from step two of the analysis. The systematic literature review was therefore extended by further monographs in order to display a solid, qualitative, and comprehensive picture of the definitions and the most current range of publications.

Based on this extensive systematic literature research, the terms corporate start-up and corporate start-up entrepreneurship are defined. The definitions rely on established definitions and new findings on entrepreneurship and intrapreneurship specified by the organizational framework of the corporate setting.

## Results

The search for English-language articles appearing in the EBSCO database published up to January 2023 in scientific journals returned 83 peer-reviewed articles which were all included in a full paper analysis to assess the relevance.

### Descriptive analysis

**Figure 3:**
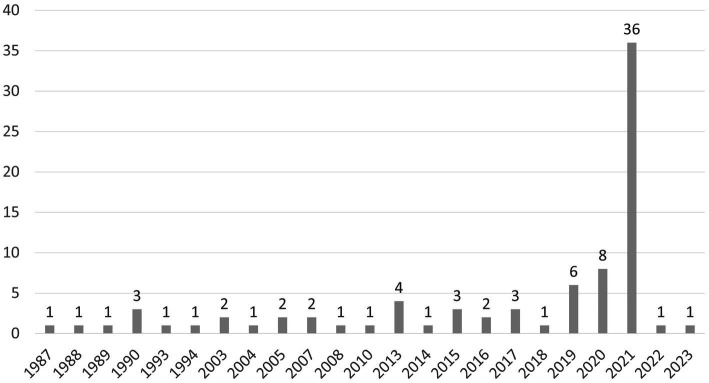
Number of publications with reference to our corporate start-up entrepreneurship (CSE) keywords per year.

To objectivize the descriptive analysis, we conducted this analysis utilizing a standardized spreadsheet, which was developed *a priori*. We gathered information regarding the author(s), title, year of publication, journal, findings, critique, and respective keyword combinations. The oldest paper dates back to 1987, and the most recent was published in 2023 (the search ended in January 2023), as shown in Figure 3. Over the years, the number of articles increased significantly, from mostly 1 to 2 articles per year in the beginning to a much larger number only recently. The articles and authors were predominantly from the fields of economics and human sciences.

The selected articles were published in 58 different journals. Sixteen of these journals published more than one article in this field (see Table 1). *Small Business Economics* was by far the journal that represented the most relevant articles (6 publications of the 83 total papers).

**Table 1:** Journals that published an article in the direction of CSE in the source title within our SLR, along with the number of articles relevant in this respect.

Journal	Number of articles
Small Business Economics	6
Entrepreneurship Theory & Practice	3
Frontiers in Psychology	3
International Entrepreneurship & Management Journal	3
Journal of Entrepreneurship in Emerging Economies	3
Strategic Management Journal	3
Foundations & Trends in Entrepreneurship	2
IEEE Potentials	2
International Journal of Innovation Management	2
International Small Business Journal	2
Journal of Management	2
Journal of Small Business & Enterprise Development	2
Management Decision	2
Psychology Research & Behavior Management	2
Review of Managerial Science	2
Sustainability	2
*Number of other journals that published an article in the direction of CSE within our SLR only once.*	*42*

Based on this extensive systematic literature research, the term corporate start-up entrepreneurship is defined. It is based on the definitions and findings on entrepreneurship and intrapreneurship specified by the organizational framework of the corporate setting “start-up niche in an enterprise,” which explains the importance of the prefix “corporate” and the distinction from intrapreneurship.

### Qualitative analysis

Our qualitative analysis is split into the sections of (1) corporate start-ups and (2) corporate start-up entrepreneurship.

#### Corporate start-ups

A comparison of the term start-up, especially with the goal of innovation, with the term split-off has shown that we need to adapt and recommend certain framework conditions so that entrepreneurship, especially ownership and risk-taking, as well as entrepreneurial motivation, can prevail. Accordingly, as a result of the comparison in column three, Table 2, we have defined corporate start-ups more explicitly.

**Table 2:** Comparison of start-up and corporate start-up framework, based on literature review.

	Start-up	Split-off	Corporate start-up
Scope of action	Entrepreneur sets off own ideas; mainly exploration (Schumpeter, 1912; Low and Mac Millan, 1988; Szyperski and Nathusius, 1999; Held, 2019)	Mostly, business ideas are followed (Kollmann, 2007; Li et al., 2020), sometimes also strategic entrepreneurship or restructuring, mostly exploitation (Szyperski and Nathusius, 1999; Jung et al., 2015)	Entrepreneur rises own idea or creates a sense of ownership for business idea; has to be exploration not solely exploitation
Risks and ownership	Risk and ownership as key attributes for true entrepreneurs (Schumpeter, 1912; Pinchot, 1987)	Management of split-off is usually still employed by the parent corporation, which limits risks and ownership (Pinchot, 1987; Li et al., 2020)	Risk and ownership lie within leader(s) of the corporate start-up
Motive	Entrepreneurial motivation (Naffziger et al., 1994; Shane et al., 2003); Desire to realize one’s own concepts and creativity, striving for greater decision-making freedom, independence, demand for higher income, existing frustration in the current workplace, lack of career opportunities or current unemployment, maintaining a family tradition, etc. (Held, 2019; Sternberg et al., 2021a,b)	Management or research and development divisions see innovative business ideas and splits-off a respective unit with their respective personnel. Personnel is not assessed or hired concerning entrepreneurial motivation; best case, employees are given a certain amount of time and budget to devote to new innovations (Pryor and Shays, 1993; Li et al., 2020)	Entrepreneurial motivation (need for achievement, locus of control, vision, desire for independence, passion, drive, goal setting, self-efficacy) and seeing business opportunities beyond the core business
Start-up life cycle	Go through the complete start-up life cycle process independently (Held, 2019)	Dependence on the parent corporation given at least as long as the split-off performs in terms of revenue and growth (Li et al., 2020)	Pre-seed and seed phases can be completed within parent corporation as long as the entrepreneur owns shares and takes risks
Legal form	All legal forms possible (Hornsby et al., 1993; Bundesministerium für Wirtschaft und Klimaschutz, 2021)	Differs by country: in the U.S., corporations (Co/Inc) are mostly represented under split-offs; in Europe, limited liability corporations (LLC) are more common (Statistisches Bundesamt, 2022; U.S. Small Business Administration, 2023)	A legal form which provides ownership of the entrepreneur, e.g., a corporation/stock corporation
Finance/resources	Employee growth comes only with rising revenue growth or funding (Chrisman et al., 1998; Song et al., 2008)	Personnel and financial resources are fully provided upfront; sometimes competition for resources among other innovative business areas (Li et al., 2020)	Personnel and financial resources might be provided to start and boost the business idea. Employee growth should come through own performance
Management and leadership	Entrepreneur (Chrisman et al., 1998; Chorev and Anderson, 2006; Kühnapfel, 2015; Malik, 2016)	Management or intrapreneurs (Li et al., 2020)	Corporate Start-up Entrepreneur
Processes	Legal and economic independence depending on legal form and ownership (Pinchot, 1987; Hornsby et al., 1993)	Full range from little co-determination to overall co-determination and monitoring (Zahra, 1996; Li et al., 2020)	Legal and economic independence
Products and services	New venture (innovation) (Schumpeter, 1912; Kollmann et al., 2021)	New venture or strategic entrepreneurship (Zahra, 1996)	New venture (innovation)
Competition/market access	Innovative business field; market is new to entrepreneur (Sandberg and Hofer, 1987; Malik, 2016)	The not core business field of the corporation; sometimes proximity to core business and customers (Li et al., 2020)	Not the core business field of the corporation
Team	The founding team knows each other through friendships or from university (Kollmann et al., 2021)	Organizational affiliations affect team composition. Sometimes whole divisions are split-off in terms of restructuring rather than entrepreneurial motivation (McKendrick et al., 2009; Li et al., 2020)	Best case, staff should be chosen in means of entrepreneurial motivation regarding the new venture business idea. Being an entrepreneur is imperative for the objective of innovation
Seniority	Younger than 10 years (Kollmann et al., 2021)	No numbers available	Younger than 10 years

##### Start-ups

There is no uniform, common definition of start-ups available, especially in the scientific field. Accordingly, for this paper and to ensure a common understanding, the following parameters are derived from our SLR and used for a definition and delimitation in this paper: The word start-up is derived from the English term “to start up something.” It is thus the symbol for the foundation of a new corporation. The Gabler Economic Encyclopedia describes start-up as a newly emerging, not yet established corporation that wants to realize an innovative business idea (Achleitner, 2018). The German Start-up Monitor shows similar attributes and defines a start-up based on the following three characteristics: younger than 10 years, highly innovative with significant employee and/or revenue growth, and the founding team mostly knows each other through university or friendships (Kollmann et al., 2021; Sternberg et al., 2021a). Furthermore, global definitions also refer to start-ups as new businesses by entrepreneurs (Low and Mac Millan, 1988). Also, while some researchers already define start-ups as the business area that describes entrepreneurial independence, others define more narrowing factors by saying that start-ups are those businesses in which new forms of business are founded, and the founders are understood as those who have significantly shaped, but also carried out, the start-up life cycle process (Held, 2019), which is associated with different characteristics, opportunities, and responsibilities. The life cycle with its six phases shows that start-ups are young corporations with innovative, scalable business ideas. They start with finding new ideas and making them feasible in the pre-seed phase, create business plans and market analyses as well as decide on the legal form in the seed phase, and preparing their product or service for the market launch in the start-up phase. In the development phase, the first sales are generated, and the business focuses on operational business capability, processes, and structures are professionalized. If the start-up continues to grow, this phase will bring a change in the team and not all founders will remain loyal to the start-up. In the final phase, start-ups orient themselves to future markets, acquisitions, and mergers (Freiling and Harima, 2019). Also, there is research rising on the topic of entrepreneurial motivation as a key success factor for start-ups. Entrepreneurial motivation is understood as those drivers of an entrepreneur that influence the direction and intensity of the entrepreneur’s activities and move him/her to a higher level of performance to achieve the goals (Naffziger et al., 1994; Shane et al., 2003). Looking at the legal form and ownership, mostly independent forms are chosen (Hornsby et al., 1993; Bundesministerium für Wirtschaft und Klimaschutz, 2021). The life cycle and definitions show how start-ups can be distinguished from new ventures; for instance, not all new ventures aim to be innovative (Kollmann et al., 2021; Metzger, 2021).

We found that the characteristic common to all definitions is the characterization of start-ups as emerging, innovative corporations that primarily pursue innovative growth objectives independently going through the start-up life cycle in whole or part.

##### Split-off

With our SLR, we gathered what is generally understood and practiced by split-offs. When a corporate group decides to split-off an entire division of a corporation or a department, some researchers and practitioners speak of corporate start-ups (Klepper and Sleeper, 2005; Jung et al., 2015). We found out that some researchers refer to either split-offs or spin-offs, differentiating whether the divestiture is in the interest of the parent corporation or not. For us, a distinction regarding the starting point as to what degree the divestiture is wanted is irrelevant. Accordingly, we refer to split-offs in the following, regardless of whether they are intentional or unintentional divestments. We found out that a majority of the ideas that lead to divestments are generated in the dedicated research and development department of a group. Corporations decide to find start-up corporations if the new business field is not part of the core field and core products but still holds promising business prospects (Kollmann, 2007; Klepper, 2009; Li et al., 2020). New ideas may not grow in the established processes of a hierarchical group and may perish, primarily if the idea is not related to the core business. Also, divestments rather than keeping the innovative business as part of the original organizational structure take place because the existing customers and the core business must continue to be served and must continue to exist, but this usually leaves little budget and personnel to deal with ideas and technologies that do not yet generate revenue at this point, but do expend resources. The desired entrepreneurship is difficult to release in established, hierarchical organizations but possible in newly created corporations (Fröndhoff, 2007). The leading team is characterized to be intrapreneurial (Stevenson and Jarillo, 1990; Li et al., 2020). In general, corporate start-ups take over employees from the parent corporation, leading to employees being only hired from the external market in exceptional cases. In our SLR, we found out that some authors say the higher the level of resources taken over and ongoing co-determination from the parent corporation, the lower the degree of freedom and scope for shaping the corporate start-up (Kollmann, 2007). Also in split-offs, personnel resources are seldom selected because the split-off employees excel in entrepreneurship. In some countries, the works council, if present in the corporation, helps shape such transformation processes and influences personnel transfers. Employees do not always have the choice of remaining in the parent corporation or joining the corporate start-up. It is instead a decision whether to be joining the split-off or terminate (Fröndhoff, 2007; Klepper, 2009; McKendrick et al., 2009; Li et al., 2020). The degree of autonomy of the split-offs differs significantly in practice. While some split-offs are entirely autonomous from the parent corporation, others generally only have legal autonomy. The economic dependency may vary and be subject to restrictions (Zahra, 1996; Kollmann, 2007; Li et al., 2020). Furthermore, the dependency of the split-off also relates to the provision of resources. In some cases, the parent corporation at least provides consultancy input; in other cases also substantially supports and co-determination, as well as provision of resources, is practiced by the parent corporation, thereby limiting the business risk of the split-off entity (Li et al., 2020). The continuing interdependence aims to profit in the different phases of the start-up life cycle from both sides. The new unit essentially benefits from the parent corporation at the pre-seed and seed phase due to reduced risk and resource provision (budget and personnel). In the course of time, the relationship tilts in the event of successful management so that technology and knowledge transfer take place bilaterally, and the parent corporation ultimately also participates in expertise, market share, and earnings/revenue. Furthermore, in some cases, the option of selling or reintegrating the split-off is also relevant for the parent corporation (Kollmann, 2007). Regarding the number of split-offs, we found out that according to a 2017 study, between 2001 and 2005, less than 10% of the corporations proposed divestitures or split-offs, while the percentage between 2011 and 2015 was already at 25% (Emrick et al., 2017). The industry sector accounts for the largest share of the split-off volume, followed by the consumer and tech sectors (Emrick et al., 2017). The legal forms mostly represented are, e.g., corporations (Co/Inc) in the United States or limited liability corporations (LLC), e.g., in Europe (Statistisches Bundesamt, 2022; U.S. Small Business Administration, 2023).

### Synthesis

The findings of our SLR indicate that a comparison of both terminologies, “start-up” and “split-off,” is essential and leads us to merge both and introduce the term corporate start-up. First of all, the tabular comparison is aligned with the start-up ecosystem model by Kollmann et al. (2021) who characterize start-ups along management and team, competition/market access, finance, products and services, and processes. We have found that corporate start-ups cannot be categorized entirely as either start-ups or split-offs, so a separate definition must be established. Typical differences can be found in terms of action, risk and ownership, motives, start-up life cycle, legal form, resources, management and leadership, processes, products and services, market, team and potentially seniority.

Thus, we define corporate start-ups as follows: *A corporate start-up is defined as a decentralized part of a corporate group with the aim of innovation and growth in a new market.* The characteristics of corporate start-ups are shown in Table 2.

We can already see differences if we look at the emergence of start-ups vs. that of split-offs. While a start-up is an original foundation that grows from its own resources, independent growth is not a given because it is usually a derivative foundation, which means that the split-off grows from existing structures and resources. The type of start-up has a decisive influence on the development of the new corporation, its goal and vision, and thus on its entrepreneurship vs. intrapreneurship and innovation (Szyperski and Nathusius, 1999). If a start-up can grow from its own resources and is less dependent because it can create more itself but also has more risk because it has full responsibility, the innovation process is less affected. Start-ups are usually founded with innovation as the main goal, while split-offs can also be created through restructuring and keeping the option open to sell the split-off if it is not successful. Innovation is not always the main goal of split-offs (Jung et al., 2015). The personal motives for founding a start-up differ greatly from those of a corporate start-up. For start-up founders, the list includes the desire to realize one’s own concepts and creativity, the pursuit of more decision-making freedom, independence, the desire for higher income, existing frustration at the current job, lack of career opportunities or current unemployment (Held, 2019) and maintaining a family tradition (Kollmann et al., 2021), to name a few. Many of these motives are not even possible in a split-off, e.g., independence from existing structures or escape from frustration in current employment. So we see that scope in the sense of ownership and risk, as well as the motive for founding, have an indispensable influence. In addition, start-ups exist in any legal form. They start with an average of 2.5 people (full-time equivalent) and the founding team knows each other through friendships or from university (Kollmann et al., 2021). Unlike start-ups, split-offs are usually founded as limited liability corporations (Europe) or corporations (U.S.). The legal form already shows that the managing directors of the split-off are restricted in their scope of action. From Schumpeter we learned that ownership, responsibility, and risk are prerequisites for entrepreneurship which is why we state that corporate start-ups need an entrepreneur who has the entrepreneurial motivation to pursue the business idea as well as ownership and risks, performing in a legal form which supports this (e.g., corporation). From a financial perspective, in most cases, there is financial dependency on split-offs. But start-up founders especially want financial independence (Kollmann et al., 2021). The financial dependencies influence the decision-making ability of the corporation owner and, thus, the processes and the extent to which the management of a start-up or a split-off determines the strategy. In start-ups, the freedom to shape the strategy is generally much greater than in split-offs. In addition, in the case of split-offs, entire divisions are also spun off. These are rarely small and consist of several employees. In contrast, the teams in successful start-ups start small and hire as they grow, which regularly brings influence and knowledge from outside into the corporation. Split-offs rarely start small and rapidly grow, thus having little claim to recruit personnel from outside. Besides, in corporate groups there is more of a staff reduction than a staff increase. This is often also transferred to the split-off. As a result, the growth in knowledge and the external perspective are sometimes entirely denied. This is why we derive that corporate start-ups should have entrepreneurs at least on leadership level and should firstly, not automatically take over entire divisions since they are part of the organization; secondly, pay attention to entrepreneurial motivation when forming the team; thirdly, make sure that hiring and external influence, as well as exchange, are possible.

An essential part of the team and a significant influencing factor on new venture performance is the corporation founder/owner. Suppose it is clear that the founder, his attitude, his experience and his leadership have an influence on success. In that case, it is also decisive whether the founder is an entrepreneur (start-up) or a manager/leader/intrapreneur who may have been active in the corporation before and was not selected based on entrepreneurial skills/motivation. That is why we contribute a separate section on corporate start-up entrepreneurship.

#### Corporate start-up entrepreneurship

To elaborate on corporate start-up entrepreneurship, we compared the definitions of entrepreneurship (in start-ups) and intrapreneurship (in corporations).

##### Entrepreneurship

According to various studies, from the early years and recently, the entrepreneur of a start-up is considered to have an essential role in the success of the firm (Schumpeter, 1934; Goldstein and Mayer, 1962; Sandberg and Hofer, 1987; Brüderl and Schüssler, 1990; Chrisman et al., 1998; Gartner et al., 1999; Chorev and Anderson, 2006; Gilbert et al., 2006; Meinhart, 2007; Storey, 2011). Some studies even identify the role of the entrepreneur as the most important driver in terms of start-up success (Goldstein and Mayer, 1962; Gartner et al., 1999; Chorev and Anderson, 2006) and innovation (Fueglistaller et al., 2012; Kuratko and Hornsby, 2021). An overview of the analyzed definitions, which shows the multilevel nature, can be found in Supplementary Tables S3a, S3b. We anticipate that we found that no definition was used more than once in the respective articles of our first SLR loop. One of the first definitions of entrepreneurship can be traced back to Schumpeter, who said that entrepreneurship concerns the whole economy and focuses on innovation. In his view, it is about combining resources in a new combination to disrupt the current state of the market. Furthermore, he was one of the first to say that entrepreneurs do not have to be self-employed, but that there are also entrepreneurs in an organization as long as they have a certain majority of shares to exercise innovation and disruption (Schumpeter, 1934). We point out that Stevenson and Jarillo (1990) essentially define entrepreneurship by saying that entrepreneurs are the people who seize opportunities and find ways to make them happen—regardless of the resources and processes for which they are responsible. With this, they confirm that entrepreneurs may exist in start-ups and corporations (Stevenson and Jarillo, 1990). Further, Czarniawska-Joerges and Wolff (1991) refer to entrepreneurship as leadership in contexts that are novel and where leaders cannot react with routine. According to them, entrepreneurship can be defined as leadership in challenging situations (Czarniawska-Joerges and Wolff, 1991). This is why we also looked at the definitions of entrepreneurial leadership and their respective characteristics. Kuratko and Hornsby (2021) elaborated on entrepreneurial literature by defining entrepreneurs as those people who master the task of identifying and developing ideas, testing them for practicability, marketability, benefits and competition, and deploying them in accordance with the resources required and adapted to the scope. This makes them innovators and catalysts for change in the business world (Kuratko and Hornsby, 2021).

To sum it up, in all definitions, there is consensus that there must be an opportunity as well as a certain degree of creation, innovation, and riskiness from the entrepreneur. Most definitions also have in common that entrepreneurs have the will to go their own way and take advantage of opportunities (Stevenson and Jarillo, 1990). Regarding the factor of resources, whether owning them or obtaining them from elsewhere, as well as organizational form, whether entrepreneurs are self-employed vs. can also be employed, there is the highest disagreement in science (Fueglistaller et al., 2012).

##### Antecedents

Supplementary Table S4 shows an overview of the antecedents mentioned by the authors in our SLR. They are clustered according to individual, organizational, and environmental antecedents and show the corresponding author behind it as well as the number of how many articles addressed the antecedent (also mentioned in the brackets below).

##### Individual

Our SLR shows that there is disagreement in science with regard to the personality traits that (should) exist in an entrepreneur. Our SLR shows that the traits which are mentioned most in the literature include risk-taking (10), innovativeness (10), vision (8), initiative (6), creativity (6), human relations (6), and business acumen (6). Also, it is extremely difficult to confirm a causal link between psychological characteristics and entrepreneurship (Stevenson and Jarillo, 1990). Much research in this area has been conducted within a framework that has been supported by public policy and is therefore not considered entirely independent. Furthermore, much literature denies the causal effect of psychological traits and entrepreneurship. Hence, we can only speak of a correlation rather than a causal effect. Another problem with the research is that too little distinction has been made between entrepreneurs as individuals and organizations. Entrepreneurship was often equated with management, which diluted the results. In other words, personal characteristics are important, but environmental and organizational variables are important, too (Stevenson and Jarillo, 1990).

##### Organizational

First of all, hierarchy levels, operational and organizational structure as well as the legal form of an organization, are one of the organizational antecedents which is mentioned the most (3) followed by leadership and management support (Urbano et al., 2022). Concerning support from within the organization, mentors, consultants, and co-founding friends/colleagues can positively affect and enable entrepreneurship but may distort decision taking and operating by emotional attitudes (Kollmann, 2007), so either direction of influence is mentioned in the articles. A third ambiguously discussed organizational antecedent is financial management and proper handling of resources: both, too little and too many resources are crucial. For instance, too much spending and thus liquidity bottlenecks or wrong spending of resources that resources are not procured, careless handling of credits can be impediments, while not investing and taking risks can be an impediment as well (Zacharakis et al., 1999; Cardon et al., 2011).

##### Environmental

Enabling factors can also concern the environment (Stevenson and Jarillo, 1990; Sitkin, 1992; Atsan, 2016; Urbano et al., 2022). Our SLR confirms that it is noticeable that through the increased use of the internet and social media, people are far more keen to exchange ideas about entrepreneurship, and teach each other, and thus, the threshold for starting one’s own entrepreneurial journey is much lower (Stevenson and Jarillo, 1990; Kollmann et al., 2021). This can also be confirmed by the numerous articles excluded from the first loop of our SLR, which focused on entrepreneurial education or cultural and gender differences. It also supports the fact that entrepreneurial education is mentioned numerous times (3) as an environmental antecedent specifically for entrepreneurship. For instance, by offering a less risky scope rather than operating on the market, universities and governmental institutions increase the opportunity for entrepreneurship (Kuratko and Hornsby, 2021). Here, too, ambiguity was mentioned concerning governmental policies that may impede entrepreneurship when they do not support start-ups, which makes it difficult for them to enter the market or challenge the process of business foundation. Also, customer proximity and market forces, e.g., stronger competition and market innovations, may affect entrepreneurship (Sitkin, 1992; McGrath, 1999; Kollmann et al., 2021).

##### Looking at intrapreneurship

The authors in our SLR emphasize the economic significance that large corporations have drawn from the conclusion that entrepreneurship may also exist in corporations (Kuratko and Hornsby, 2021). We found that interest in intrapreneurship goes back to the 1980s (Schollhammer, 1981; Pinchot, 1985, 1987) and is therefore not a new field of research, but it has not yet been scientifically fully explored as most papers mention in their conclusion. Furthermore, the state of research is very fragmented, and the scope of when to speak of intrapreneurship and when not is very diversely presented. Concerning the scope of intrapreneurship, Schumpeter already includes activities of individuals who are not merely self-employed; this paper pursues the general understanding that intrapreneurs are employees, which limits their scope of responsibility and ownership and limits innovation (Schumpeter, 1934; Wennekers, 2006). Wennekers (2006) distinguishes entrepreneurs from intrapreneurs based on the degree of employment (self-employed vs. employee; first level) and a behavioral dimension (second level). Either a person exhibits entrepreneurial behavior, which is self-initiated and aims to identify, exploit and implement opportunities. Or the person exhibits behavior that relates to the coordination and organization of resources and is, therefore, more managerial behavior. According to Wennekers, only those who are employed and who exhibit entrepreneurial behavior in this setting are intrapreneurs (Wennekers, 2006). It shows that it depends on both the degree of employment and the individual’s behavior, whether s/he is an intrapreneur in a corporation and that the degree of intrapreneurship of a corporation depends on the sum of the individuals. Moreover, Stevenson and Jarillo (1990) also focused on the behavioral aspect in their definition. They described intrapreneurship as the intrinsically motivated, innovation-oriented, and entrepreneurial actions of employees in an organization as if they were entrepreneurs. Whether intrapreneurship can exist in corporations depends to a large degree on the extent to which the employees below the management level also have an entrepreneurial attitude and (are able to) practice it (Stevenson and Jarillo, 1990).

#### Antecedents

##### Individual

Our SLR shows that here, too, is disagreement in science with regard to the traits of an intrapreneur. We found that the traits that are mentioned most in the literature include being innovative (9), opportunity oriented (6), having entrepreneurial qualification or experience (5), and being able to create a vision (5). Concerning the development of research on individual antecedents, we found that Hornsby et al. (1993) reacted to the research gap that until then, preconditions for intrapreneurship were only examined from an organizational perspective. They supplemented the existing scientific findings with individual characteristics and stated risk-taking propensity, desire for authority, need for achievement, goal orientation, and internal locus of control as the most mentioned and most relevant individual antecedents for intrapreneurship (Hornsby et al., 1993). Our SLR showed that other characteristics were mentioned more often (see above). We also found that the prerequisites mentioned in the model by Hornsby et al. (1993) need to be supplemented by other perspectives that now exist. Especially entrepreneurial experience and qualification increases in being addressed by authors as well as connecting the dots in strategic, future-oriented thinking and seeing the impact of an opportunity are skills that already must be present in the employees or trained when it comes to intrapreneurship in contrast to entrepreneurship (Stevenson and Jarillo, 1990). Some authors go beyond entrepreneurial skills and mention entrepreneurial motivation, meaning the desire to establish a business, the recognition of opportunities, and the sustainability with which they are realized, as a prerequisite for intrapreneurship (Naffziger et al., 1994; Shane et al., 2003).

##### Organizational

Much research has been carried out on organizational obstacles and conducive factors (Schollhammer, 1981; Pinchot, 1985, 1987; Hornsby et al., 1993; Pryor and Shays, 1993; Antoncic and Hisrich, 2001; Cannon and Edmondson, 2005). Our SLR indicates that resource provision (7), independence rather than bureaucracy and monitoring processes (6), as well as management commitment (6), are the most mentioned antecedents referred to intrapreneurship. Commitment is often also mentioned along with trust and integrity of the management in order to facilitate entrepreneurial work in a corporation. For instance, managers must represent and exemplify intrapreneurship, approaching new paths, being able to fail, and contributing to growth by innovation with credibility. Some articles emphasize the need for sponsors who, on the one hand, have the influence and power to make decisions and provide the dedicated resources, so being on the management/leadership level, and on the other hand, who listen to the intrapreneurs and have the time to deal with them, coach and accompany them without interfering (Pryor and Shays, 1993). When intrapreneurs are constrained within the corporation, either by management or processes, they are initially angered, may vocalize their displeasure, or leave the corporation and go to competitors or start their own corporation, thus competing with the corporation they once belonged to. This also includes independence for the intrapreneur in decision-making processes (Kanter, 1983; Pinchot, 1985, 1987; Hornsby et al., 1993; Pryor and Shays, 1993). A relevant finding concerning the context to split-offs is that further papers indicate that people cannot be appointed or convinced to become intrapreneurs, but they have to carry these characteristics within themselves. Accordingly, it is important not only to look for topics and innovations in the corporation, but also for employees who already distinguish themselves as intrapreneurs (Pryor and Shays, 1993). Also, with regard to split-offs, we found out that organizational structure is often mentioned as crucial but with different implications, for instance, differentiating between new venture entrepreneurship or strategic entrepreneurship (Hornsby et al., 1993; Neessen et al., 2019). Nevertheless, the type of organization and in-depth study of their impact has so far remained neglected in the literature (Belousova et al., 2020), and our SLR strengthens the background of the paper to examine intrapreneurship in the niche of corporate start-ups more closely as it represents a special context. The organizational structure is of unique relevance as, e.g., some antecedents of entrepreneurship are *per se* excluded and cannot be artificially created, e.g., the motive to create a business due to continuing family businesses or out of necessity. In organizations, the urgency is not necessarily high due to the payroll, such as in Germany, due to works constitution law and other employee-friendly measures (Stevenson and Jarillo, 1990). Since splitting-off is a decision concerning the corporation’s strategy, we would like to highlight that we found out that too much and too sudden confrontation with change can harm intrapreneurship as well as many personal changes in the management or when dismissals are carried out by lower levels of management as a pawn sacrifice (Kanter, 1983). Also, like entrepreneurs, it is crucial that employees perceive recognition of intrapreneurship to intensify the sense of ownership, e.g., through intra-capital, to set positive incentives so strongly that the motivation to create, see opportunities, and implement them is increased. Successful intrapreneurs should use intra-capital for (re-)investments in other projects so they become inter-venture capitalists (Pinchot, 1987; Stevenson and Jarillo, 1990).

##### Environmental

Various papers in our SLR mentioned customer proximity (5) as important as being aware of the market (5) (e.g., product addresses market needs, intrapreneur follows market trends) as an antecedent for intrapreneurship because it is the customers and the market who give quick and valuable feedback, counteracting getting tied up in internal processes and bureaucracy. Also, communities to network are not only internally relevant but also externally in order to inspire and create synergy effects (Pryor and Shays, 1993).

### Synthesis

The findings of our SLR indicate that a comparison of both terminologies, “entrepreneurship” and “intrapreneurship” is indispensable, which is why we made the tabular comparison in Table 3. We found that both terms differ in scope, goal, focus, resource availability, dependencies, external influences, time constraints, risk, and profit. Again, this shows that we need a definition for corporate start-up entrepreneurship, which also not yet exists. Our definition of corporate start-up entrepreneurship fits the setting of corporate start-ups (see above) and is based on the detailed definition of an entrepreneur by Kuratko and Hornsby (2021), who define “entrepreneurs [as] those people who master the task of identifying and developing ideas, testing them for practicability, marketability, benefits and competition, and deploying them under the resources required and adapted to the scope; this makes them innovators and catalysts for change in the business world” (Kuratko and Hornsby, 2021, p.5). We have deliberately chosen this definition among the many available because it is detailed, includes several characteristics, distinguishes between having ideas and also implementing ideas, says that the impact of innovation affects the business world in general, and does not exclude the framework that entrepreneurship prevails only in start-ups. Moreover, it is a novel entrepreneurship definition that results from a literature review reflecting developments in recent years. It was also important to us that the definition of corporate start-up entrepreneurship should make it clear that the corporate start-up entrepreneurs are the ones who bring innovation into the parent corporation and not the divisions of the core business that are not spun off. The original definition provides a good basis for specification. Thus, we derive:

**Table 3:** Deriving “corporate start-up entrepreneurship” from entrepreneurship vs. intrapreneurship comparison (own representation based on a literature review).

	Entrepreneurship	Intrapreneurship	Corporate start-up entrepreneurship
Scope	Self-employed and sets of own entrepreneurial ideas (Wennekers, 2006; Fueglistaller et al., 2012); mostly exploration	Employed and sets of entrepreneurial rather than managerial ideas; mostly ambidextrous (Schollhammer, 1981; Pinchot, 1987; Antoncic and Hisrich, 2003; Wennekers, 2006)	The corporate entrepreneur of CSE shall eventually be employed by the corporate start-up, holding a respective amount of ownership and responsibility (at least 20%). S/he may start the pre-seed and seed phase in the parent corporation but proceed with subsequent phases mandatorily in corporate start-up, therefore unavoidably not being employed by the parent corporation anymore; business concept shall be explorative rather than exploitative
Ownership of concept and ideas	Entrepreneur (Kuratko and Hornsby, 2021)	Intrapreneur is employed; therefore, a corporation is true owner of concepts and ideas (Wennekers, 2006)	Ownership shall be with the CSE, e.g., by shareholding, organizational structure as an independent corporation, etc.; there needs to be financial liability
Resources	Mainly provided by entrepreneur, partly by investors (Fueglistaller et al., 2012; Kuratko and Hornsby, 2021)	Provided by the corporation (Pinchot, 1987; Stevenson and Jarillo, 1990; Hornsby et al., 1993; Pryor and Shays, 1993)	In pre-seed phase and seed phase, CSE shall be given a certain amount of time (e.g., 20% innovation time) and resources besides day to day work to be able to work on innovation.
			In subsequent phases, as of start-up phase, resources shall be provided to a certain degree by the parent corporation with fixed time-frame and goal-orientation.
			Furthermore, being part of a split-off team/division should not mean that one is automatically allocated to the corporate start-up. Personnel resources should result from entrepreneurial motivation and not from historical, organizational affiliations; recruiting shall include CSE checks
Focus of work	Entrepreneurs develop innovation through either new knowledge, new services new processes, new products, or new markets (Schumpeter, 1934; Gartner, 1988)	Intrapreneurs work is not necessarily innovate sometimes rather strategic entrepreneurship, meaning restructuring; mainly entering the existing market (Hornsby et al., 1993; Neessen et al., 2019)	CSE shall work on new venture and innovation rather than strategic entrepreneurship (restructuring/rejuvenation etc.) of the parent corporation in the existing market
Dependencies and decisions	Entrepreneur acts autonomously; create, act and decide upon own target/vision (Fritsch, 2020; Kuratko and Hornsby, 2021)	Intrapreneur works within existing system (non-autonomously); act upon set goals/vision and have restricted freedom to design (Wennekers, 2006; Maier and Pop Zenovia, 2011)	CSE acts autonomously in corporate start-up
External influences	Entrepreneur is susceptible to outside influences, be it positive or negative (Stevenson and Jarillo, 1990; Kollmann, 2007; Kuratko and Hornsby, 2021)	Intrapreneur and corporation are less susceptible to external influences (Pryor and Shays, 1993)	CSE shall build up network internally (parent corporation) and externally (market)
Risk	Entrepreneur takes all risks (Schumpeter, 1934; Kollmann et al., 2021)	Corporation takes risks (Stevenson and Jarillo, 1990)	CSE and parent corporation share risks according to distribution of share
Time constraints	Pressure to show quick results and success (Kuratko and Hornsby, 2021)	Moderate pressure to show quick results (Pryor and Shays, 1993)	Defining of certain time frame in pre-seed phase among shareholders shall determine time constraints
Compensation/profit	The entrepreneur gets compensation according to ownership (Zacharakis et al., 1999; Cardon et al., 2011; Kollmann et al., 2021)	Limited compensation for intrapreneurs (Pinchot, 1987; Stevenson and Jarillo, 1990)	CSE shall not be paid fixed salary but a variable salary according to corporate start-up performance
Failures	Little flexibility: there is no sanction by superiors (possibly just by investors), but there might be instant existential threats (Sternberg et al., 2021a,b)	Dependent on culture of failure, a single mistake can lead to substantial consequences (e.g., withdrawing from project) but also more flexibility in staying with the corporation and balancing out smaller mistakes (Kanter, 1983; Maier and Pop Zenovia, 2011)	Failures of the corporate start-up entrepreneur may lead to dissolving the corporate start-up and losing ownership; yet, the CSE shall be supported by the parent corporation to apply his entrepreneurial skills within the internal or external network/projects for a certain period of time (e.g., fallback to parent corporation no longer than 3 month)
Challenges	Novel contexts, market know-how and acceptance, credibility (Czarniawska-Joerges and Wolff, 1991)	Corporate culture, politics, processes and regulations limit independence in operation (Kanter, 1983)	Balance between co-determination among owners; setting the framework (target, budget, etc.) upfront
Effect on innovation	Entrepreneurship as most important driver for innovation (Schumpeter, 1934; Fueglistaller et al., 2012; Kuratko and Hornsby, 2021)	Effect of intrapreneurship depends on whether organization is suppressing innovational processes, e.g., by silo-thinking, limiting risk-taking, blaming culture etc. (Kanter, 1983; Maier and Pop Zenovia, 2011) and ownership, e.g., through intra-capital for the intrapreneur (Pinchot, 1987)	Corporate start-up entrepreneurship shall drive innovation; no restructuring purposes

*Corporate Start-up Entrepreneurship means leading a decentralized part of a corporate group with the aim of innovation and growth in a new market (=corporate start-up), i.e., to identify and developing ideas initially in the frame of the parent corporation, testing them for practicability, marketability, benefits, and competition, and deploy them in accordance with the resources required provided by the parent corporation and adapted to the given scope, existing culture and prevailing politics. This makes corporate start-up entrepreneurs the innovators for the parent corporation for the previously defined field of the corporate start-up and catalysts for change in order to continue to drive the group's competitiveness through the modified economic structure and to achieve innovation from its own resources.*

For instance, Table 3 shows that there are significant differences in terms of decision-making (autonomous vs. dependent), the goal (innovative vision vs. exploring existing markets), the handling of risks, liability, and personnel and financial bottlenecks. Intrapreneurship is not the same as entrepreneurship in corporate start-up settings. If intrapreneurship were simply entrepreneurship in a corporate setting, according to the literature review, leaders and employees would always have to proactively identify opportunities and work to generate growth and innovation. However, the review shows that dependencies on the corporation make it impossible to freely decide the vision, the product or the day-to-day decisions and to leverage resources so that innovation can grow. Thus, in order to leverage a corporate start-up to bring innovative business models to the market, independence from the parent corporation and ownership are required. This can be initiated by a suitable legal form for the corporate start-up, e.g., by shareholding. Furthermore, the fact that entrepreneurs always have to deal with uncertainty and that this drives their creativity and innovation is also not given in intrapreneurial settings, e.g., corporate start-ups. The sense of uncertainty and risk must also be created for corporate start-ups so that innovation-oriented behavior can take place. This can be achieved not only through the legal form, but also through financial liability, explorative business models and filling positions with corporate entrepreneurs instead of transferring employees from the parent corporation to the split-off. Intrapreneurship is also not entrepreneurship in corporate start-ups, because the compensation is mostly consistent, whether positively remuneration or negatively, when making mistakes. Executives and employees in split-offs remain on the payroll, they are not personally liable, and they do not fall into risks—at most, the compensation is bonus-related, as in the case of salespeople. The corporate entrepreneur must expect a loss of employment in case of mistakes as well as experience profit when the business model is successful. Thus, corporate start-up entrepreneurship requires that the corporate entrepreneur also experiences financially whether the business model is successful or not, for example, through a variable compensation model. In general, the intrapreneur can fall back on the group structures, find employment there again and not have to reckon with loss of employment. In addition, it was generally assumed that the managers and employees in the intrapreneurship setting also exhibit an entrepreneurial type of behavior. Suppose one goes by Wennekers’ rudimentary comparison of entrepreneurship to intrapreneurship. In that case, this shows that just because an employee is no longer self-employed, that does not make him an intrapreneur. If this employee were to exhibit managerial behavior, then s/he would be an executive manager, not an intrapreneur and still far from being an entrepreneur. Entrepreneurs act freely out of their entrepreneurial motivation. This would also be a desirable state for intrapreneurs, because people cannot be appointed to become entrepreneurs; they must already have the essential characteristics. Here, too, are significant differences, especially if you look at the framework of a corporate start-up. It has been shown that a corporate start-up is characterized by the fact that entire areas are spun off as the definition of split-off states (Jung et al., 2015). Those who are employed in the area at the time of the split-off are usually also spun off no matter what. However, this does not mean that these people demonstrate entrepreneurial behavior, motivation, or skills. Thus, for corporate start-ups, it is essential to employ employees with entrepreneurial motivation and skills (=corporate entrepreneurs). In general, the question arises as to what extent entrepreneurial motivation/skills exist and are promoted in corporations so that corporations can leverage them when setting up new corporations. The differences show that the desired state in a corporate start-up cannot arise as long as intrapreneurship prevails.

As a synthesis, for corporate start-ups to realize innovation for large corporations, there needs to be (1) the appropriate legal form, which ensures ownership but also the risk of the leaders, (2) an explorative focus rather than exploitation, (3) variable compensation rather than fixed and (4) corporate entrepreneurs rather than employees and managers. This can also be seen in the tabular comparison and derivative of corporate start-up entrepreneurship in Table 3.

## Conclusion

Our research focused on the research question of how entrepreneurship may exist in the niche of corporate start-ups. The result of our work shows that entrepreneurship can exist in the form of corporate start-up entrepreneurship in split-offs, provided certain antecedents and requirements are given, and there is a uniform understanding of the framework. Through a systematic literature review we have defined both the term corporate start-up and the term corporate start-up entrepreneurship, which have not yet been defined uniformly and scientifically reliable. The definition of corporate start-up entrepreneurship includes different aspects of entrepreneurship as well as a reflection of the corporate setting. Besides the definition, we deduced antecedents and impediments for corporate start-up entrepreneurship to guide corporations when splitting off divisions to pursue growth. This review confirms the strategic relevance of corporate start-up entrepreneurship concerning corporate transformations. Our SLR contributes to current academic knowledge by showing the following: Overall, we endorse the adapted conceptual model of corporate entrepreneurship by Urbano et al. (2022) as we confirm that the organizational setting (e.g., legal form along with degree of ownership, decision-making processes, resource availability and size) has an impact on entrepreneurship. The identified key antecedents for corporate start-up entrepreneurship through our SLR, (1) appropriate legal form, (2) exploratory business, (3) variable compensation and (4) corporate entrepreneurs can be assigned to the organizational and individual antecedents of the model. The degree of innovation (how far one deviates from more experienced business models and takes the respective risks) also influences whether we speak of entrepreneurship (innovative ideas) or intrapreneurship (translating ideas of others) or pure managerial behavior (consistency and order instead of change and movement). Remuneration and motivation are also critical cornerstones that determine whether a parent corporation can enable corporate entrepreneurship (when the corporate entrepreneur owns the business and the idea) or really just management/intrapreneurship in corporate start-ups (when the management of a corporate start-up acts merely on behalf of a corporation). Also, most of the current definitions of entrepreneurship and intrapreneurship assume that there is an either-or logic. Nevertheless, we showed that entrepreneurship is rather considered a continuum. A special form, which lies somewhere between entrepreneurship and intrapreneurship but arguably closer to entrepreneurship, is called corporate start-up entrepreneurship, which we have explored in this paper. We assume that other niches also need a special consideration of entrepreneurship, which could be taken into account in future research scenarios. Further future research that we recommend is, in particular, the quantitative study on corporate start-up entrepreneurship conditions based on this work. Further research could also be carried out here (e.g., a case study), to what extent corporations consider the general conditions in practice for split-offs or to what extent corporate start-up entrepreneurship prevails in corporate start-ups. There may be country-specific differences in the case of split-offs from corporations. Further future research topics could address the relation between corporate start-up entrepreneurship and corporate (start-up) performance or in-depth studies considering the relationship between corporate start-ups and the corporate group. Also, for practitioners, it could be of future interest how corporate groups prepare their employees (e.g., via trainings, assessments) and the processes (e.g., placement) regarding corporate start-up entrepreneurship. Here the antecedents could give a guiding structure. Since there was limited literature on the environmental factors available and incorporated in our SLR, future research could deepen the impact of environmental factors on corporate start-up entrepreneurship. As with all studies, our review is subject to some limitations but also offers opportunities for further research. First of all, it is possible that we missed some high-quality papers in the field of entrepreneurship in corporate start-ups. This may be because our first loop of SLR was reduced to the database EBSCO only, and our second loop, snowball systematic analyzing the references of our first loop, focused on those indices. Additionally, we did not include papers that were not written in English or German. We deliberately chose EBSCO and took our keywords derived from theoretical considerations in order to focus our research, but the linguistic limitation was not a prerequisite but unavoidable. Nevertheless, we strongly feel that due to our two-fold approach, SLR via EBSCO and the snowball approach via EBSCO and Google Scholar, we included a sufficient number of articles in our research, especially since the niche area of corporate start-ups has not yet been researched well. Despite the mentioned limitations, our results of the SLR and especially our definitions of the research gap of corporate start-up entrepreneurship serve as a solid basis for further research in this niche area.

## Author contributions

CK: Conceptualization, Data curation, Formal analysis, Investigation, Methodology, Project administration, Validation, Visualization, Writing – original draft. C-CC: Conceptualization, Formal analysis, Methodology, Project administration, Resources, Supervision, Writing – review & editing.

## Funding

The author(s) declare that no financial support was received for the research, authorship, and/or publication of this article.

## Conflict of interest

The authors declare that the research was conducted in the absence of any commercial or financial relationships that could be construed as a potential conflict of interest.

The author(s) declared that they were an editorial board member of Frontiers, at the time of submission. This had no impact on the peer review process and the final decision.

## Publisher’s note

All claims expressed in this article are solely those of the authors and do not necessarily represent those of their affiliated organizations, or those of the publisher, the editors and the reviewers. Any product that may be evaluated in this article, or claim that may be made by its manufacturer, is not guaranteed or endorsed by the publisher.
